# Oxidative Stress in Cardiovascular Disease and Comorbidities

**DOI:** 10.3390/antiox11081519

**Published:** 2022-08-03

**Authors:** Guillermo Zalba, María U. Moreno

**Affiliations:** 1 Department of Biochemistry and Genetics, University of Navarra, 31008 Pamplona, Spain; 2 IdiSNA, Navarra Institute for Health Research, 31008 Pamplona, Spain; 3 Program of Cardiovascular Diseases, Center for Applied Medical Research, University of Navarra, 31008 Pamplona, Spain; 4 CIBERCV, Centro de Investigación Biomédica en Red Cardiovascular, Instituto de Salud Carlos III, 28029 Madrid, Spain

Reactive oxygen species (ROS), both as second messengers and as contributors to oxidative stress, play a major, complex role in the initiation, development and outcomes of cardiovascular diseases. The molecular, cellular and histological alterations that ROS elicit in the heart and the vessels are intricately linked to cardiovascular comorbidities such as metabolic syndrome, diabetes, renal disease and ageing. Current therapeutic approaches aimed at alterations related to ROS imbalance lack precision, which in part explains their limited success. A better knowledge of the underlying mechanisms by which oxidative stress contributes to cardiovascular and related diseases is essential for the early, precise diagnosis and the personalized, safe, effective treatment of these diseases, leading causes of global mortality and major contributors to disability worldwide [1]. In this Special Issue, global investigators provide novel experimental evidence, as well as timely reviews, of diverse mechanisms by which ROS contribute to cardiovascular disease and comorbidities.

There is wide agreement on the relevance of endothelial alterations in the diverse phenotypes that concur in cardiovascular diseases. In this sense, growing evidence points to NADPH oxidase activity, and more specifically that of NOX5, as a key player in endothelial dysfunction. Using a conditional knock-in NOX5 mouse model, Cortes et al. [2] investigate its role in promoting ageing-related vascular alterations that lead to blood–brain barrier (BBB) damage and cognitive impairment. Even with no blood pressure effect, the authors observed that aged mice expressing NOX5 in their endothelium present reduced tight-junction protein expression, which is accompanied by the poor performance of memory tests, suggesting that vascular NOX5 may favour behavioural changes in ageing through alteration in the BBB by oxidative stress.

With a similar approach, that is, an endothelial conditional NOX5 knock-in mouse model, but in the context of obesity, García and colleagues [3] present novel findings of the potential protective effect of NOX5. NOX5 promoted the expression and activation of thermogenesis and lipolysis pathways in the mesenteric and epididymal fat of mice fed with a high-fat diet. This activation was derived from an increase in IL-6 production because of NOX5 activity, corroborating in vitro studies with 3T3-L1 adipocytes cultured with conditioned media of endothelial NOX5-expressing bEnd. Three cells treated with glucose and palmitic acid also showed IL-6 production and the activation of thermogenesis and lipolysis through phosphorylation of STAT3 and AMPK. The authors suggest that controlled endothelial NOX5 ROS production may contribute to lipid homeostasis in the neighbouring adipose tissue.

Additionally, in the context of endothelial dysfunction, Lee et al. [4] explore the role of the mitoribosomal gene CR6 interacting factor 1 (CRIF1) in the regulation of homocysteine levels in vitro and in vivo. CRIF1 downregulation resulted in increased intracellular and circulating homocysteine levels, paired with impaired dihydrofolate reductase (DHFR) activity and reduced folate intermediate levels. This phenotype was not rescued by folic acid supplementation, which may in part explain folate supplements’ limited effects. However, DHFR overexpression resulted in decreased homocysteine accumulation in CRIF1-deficient endothelial cells, which suggests that DHFR may be a potential therapeutic target in homocysteine-related diseases.

Considering the vessel as a whole, Sánchez-Infantes et al. [5] investigate mechanisms involved in abdominal aortic aneurism (AAA), as well as potential diagnostic biomarkers related to oxidative stress and inflammation to assist in its management, as it is a major cause of death in the elder population. The authors study human AAA samples and detect extensive inflammatory infiltration and increased levels of ROS. In addition, both circulating and tissue levels of inflammatory molecules are present in AAA patients, with IgG, CD38 and GDF15 showing a potential diagnostic value. These molecules could be markers as well as mediators in AAA and could complement current imaging techniques for precise risk assessment in these patients.

Regarding research on the myocardium, Valls-Lacalle and colleagues [6] explore the contribution of lysyl oxidase (LOX) to myocardial oxidative stress following ischaemia–reperfusion in a transgenic mouse model of human LOX cardiac overexpression. Under basal conditions, LOX transgenesis is associated with higher cardiac superoxide levels. However, a comparable acute cardiac ischaemia–reperfusion injury was observed in LOX-overexpressing and control mice, with similar changes in cardiac dimensions and function 28 days after myocardial infarction, suggesting that the ROS induction by LOX may be surpassed by the oxidative damage caused by ischaemia–reperfusion.

In the context of myocardial damage in ischaemic heart disease, Tarazón et al. [7] explore telomere homeostasis and its relationship with oxidative stress as a novel potential mechanism and biomarker. Through RNA-seq studies of left ventricular samples of explanted hearts from ischaemic cardiomyopathy patients and control subjects, the authors observed a dysregulation of the shelterin and cohesin complexes, associated with an increase in the response to cellular oxidative stress. In addition, the authors detect alterations in the expression of telomeric DNA repair systems, including lnRNAs involved in telomere protection in response to stress TERRA and GUARDIN, whose regulation was in turn related to ROS-depleting superoxide dismutase and catalase. Their results indicate that an altered telomere homeostasis could be a relevant consequence of oxidative stress in ischaemic cardiomyopathy.

In a population study, Sołtysik and collaborators [8] investigate the circulating oxidant and antioxidant potential and its association with the cardiometabolic risk in ageing patients. The authors assess multiple cardiovascular risk factors as well as the total antioxidative status (TAS) and total oxidative status (TOS), identifying that TAS and TOS are largely related to modifiable cardiovascular risk factors such as body mass and are affected by drugs such as angiotensin II receptor blockers. Circulating oxidation markers appear to be particularly associated with glucose concentration. The data presented indicate that there are strong connections between cardiovascular risk factors and redox potential and specify how cardiometabolic interventions may counter-balance oxidative stress.

Finally, two reviews are included in this Special Issue. On the one hand, Pothen and Balligand [9] explore the interesting concept of legacy, the sustained beneficial effect of a given treatment on disease outcomes, even after cessation of the intervention, in relation to cardiovascular risk factors. The authors reviewed clinical trials that revealed a legacy effect in relation with metabolic disease, diabetes, hypertension and hypercholesterolemia. In addition, they summarized key data from basic research pointing to potential pathophysiological mechanisms of legacy effects, based on previous studies on metabolic memory.

On the other hand, Szyller et al. [10] review the literature on the use of antioxidants in cardiac arrhythmia treatment. The authors consider that the beneficial effects of antioxidants make them interesting therapies not only as direct ROS scavengers but also in protecting against cardiac remodelling, contributing to maintaining normal cardiac function. As available evidence is mostly based on in vivo and in vitro research, there is a great need for clinical trials, both in patients with chronic arrhythmias and also in emergency and critically ill patients, where oxidative stress is very pronounced and constitutes a relevant therapeutic target.
